# Preliminary report: neural firing patterns specific for Meniere’s disease

**DOI:** 10.1186/s40463-014-0052-4

**Published:** 2014-12-20

**Authors:** Brian Blakley, Zeinab A Dastgheib, Brian Lithgow, Zahra Moussavi

**Affiliations:** Department of Otolaryngology - Head and Neck Surgery, University of Manitoba, GB421 - 820 Sherbrook Street, Winnipeg, Manitoba R3A 1R9 Canada; University of Manitoba, Room E3-512 Eng. Bldg., Biomedical Engineering, 75A Chancellor’s Circle, Winnipeg, MB R3T 5 V6 Canada; Monash Alfred Psychiatry Research Centre, University of Manitoba, and Research Affiliate of Riverview Health Center, EVestG Research Lab, Riverview Health Centre, Room PE446, 1 Morley Avenue, Winnipeg, MB R3L2P4 Canada; Biomedical Engineering, University of Manitoba, and Research Affiliate of Riverview Health Center, Room E3-513 Eng. Bldg., Biomedical Engineering, 75A Chancellor’s Circle, Winnipeg, MB R3T 5V6 Canada

**Keywords:** Meniere’s disease, EVestG, Vestibular response, Classification, Fractal dimension

## Abstract

**Objective:**

To describe the application of a new, objective diagnostic test for Meniere’s disease.

**Introduction:**

Electrovestibulography (EVestG) is a complex, newly-developed test paradigm that searches for neural firing patterns that may be diagnostic for particular neural disorders. EVestG system was previously “trained” to distinguish Meniere’s disease from other patients on a set of training data. In this paper we illustrate its diagnostic application in a new group of unknown subjects.

**Setting:**

Collaborative Academic Bioengineering Research Centre.

**Study design:**

Prospective, blinded human Clinical Trial.

**Methods:**

In an attempt to understand the specific neural firing patterns that may objectively characterize latent Meniere’s disease, two hundred fifty-six consecutive patients who presented for electronystagmography testing were asked to undergo EVestG testing. Ten subjects actually completed testing but data were too noisy to permit analysis for one patient. Complete data were available for nine patients with either a clinical diagnosis of either Meniere’s disease (4 patients) or some other vestibular disorder (2 vestibular neuritis, 2 benign positional vertigo and 1 non-specific dizziness). None of the patients were experiencing attacks of vertigo within a week of EVestG testing. Ten normal control subjects with no history or symptoms of ear disease were also tested. EVestG was performed in a separate engineering research facility by investigators who were unaware of their clinical diagnosis. If EVestG suggested that the probability of Meniere’s disease was 0.5 or greater Meniere’s disease was considered present by the objective testing. The objective and clinical diagnoses were compared.

**Results:**

EVestG testing correctly identified three of four Meniere’s disease patients and rejected the diagnosis in 9 of the 10 controls. Two of the 5 dizzy, non-Meniere’s patients were incorrectly identified as Meniere’s disease. The sensitivity and specificity of EvestG testing were 75% and 80%, respectively. EVestG results were statistically significantly different for Meniere’s patients versus the other dizzy patients and controls (Univariate ANOVA difference contrasts *p* = 0.0340) even in this small sample.

**Conclusion:**

The EVestG protocol appeared to show promise as an objective, diagnostic test for Meniere’s disease, but our sample size is too small to generalize widely.

**Level of evidence:**

N.A. Prospective Human clinical trial.

## Introduction

Meniere's disease is an inner ear disorder that can cause episodes of vertigo, ringing in the ears (tinnitus), a feeling of fullness or pressure in the ear, and fluctuating hearing loss affecting 0.2% of the population of the United States [1]. Although the symptoms can be significantly disruptive the specific electrophysiological firing patterns that characterize the disorder are unknown.

We hypothesize that a neurotologic disorder that causes marked symptoms has some underlying neural firing patterns that are unique to and diagnostic of that disorder. Currently the clinical diagnosis of Meniere’s disease is based on the clinical history often using the criteria established by the American Academy of Otolaryngology-Head and Neck Surgery (AAO-HNS) [2]. Unfortunately clinical descriptions vary across time, examiners and within the same patient. Definitive histologic confirmation of clinical diagnosis can only be made post-mortem. There is a need for objective testing to confirm Meniere’s disease specifically and vestibular problems in general.

A new test of vestibular function called EVestG [3,4] has been described that utilized sophisticated mathematical techniques to a vestibular-evoked signal that may be specific for Meniere’s disease. While many vestibular tests cause nausea and dizziness or may take prolonged time these are not issues with EVestG. This test takes about 30 minutes, involves slow, easily tolerated motion. EVestG is easier for patients to tolerate than any current clinical vestibular test. The algorithm has been trained with subjects from Alfred Hospital in Melbourne, Australia. Here we present the blinded application of the result to a new set of unknown patients from Winnipeg, Canada.

## Methods

Two hundred fifty-six consecutive patients, who presented for electronystagmography (ENG), were asked to participate in this research study. Ninety-one patients indicated that they would participate in this project but only 10 actually consented and completed all testing including EVestG, and met our other inclusion criteria (previously undergone audiometric testing and MRI scan). Thus, the database of this study included those 10 patients. Patients were not selected on the basis of any particular diagnosis. Ten age-matched normal controls were also recruited for comparison selected from staff, students and friends. Control patients denied dizziness or hearing problems. The numbers of patients approached for testing and who finally were tested illustrates the difficulties in subject recruitment in this type of research. In order to determine whether differences in EVestG results merely reflected ENG or auditory abnormalities, ENG data consisting of the sum of the four caloric exams and the unilateral weakness as well as the average speech reception threshold (SRT) were collected for the patient group.

**Figure 1:**
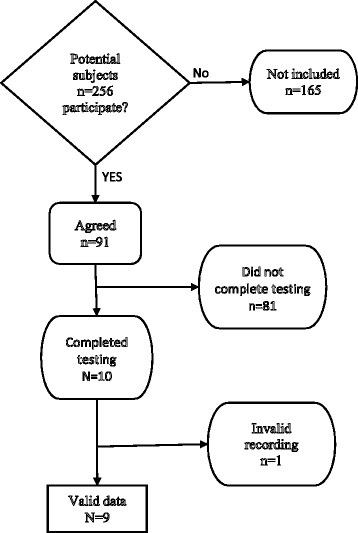
**Flow chart for recruitment of dizzy patients for the study.** In addition to the dizzy patients 10 normal controls were tested.

EVestG data for one of the 10 patients had too much electrical noise to permit analysis; thus, our final dataset consists of 9 patients including 4 with Meniere’s disease and 5 with other vestibular disorders (2 vestibular neuritis, 2 benign positional vertigo and 1 “non-specific” dizziness (see Figure 1) and 10 controls. Patients and controls were asymptomatic at the time of testing. All patients were seen by an experienced, fellowship-trained clinical neurotologist for treatment of dizziness, who assigned a diagnosis of either “Meniere’s disease” using the AAO criteria [2] or “non-Meniere’s.” ENG and audiometric findings and the diagnosis were not communicated to the researchers performing EVestG testing in another facility on another day. The protocols were approved by the Ethics Review Boards of the University of Manitoba and Riverview Heath Centre in Winnipeg.

The clinical diagnosis was benign positional vertigo in 2 patients, vestibular neuritis in 2 patients, nonspecific dizziness in 1 patient and Meniere’s disease in 4 patients. One Meniere’s patient had crisis of Tumarkin, and had undergone gentamicin ablation. One vestibular neuritis patient met criteria for bilaterally reduced caloric responses, and one of the benign positional vertigo patients had some lacunar infarcts on MRI scanning.

### Definitions of clinical diagnoses

Meniere’s disease was diagnosed in subjects who met the AAO-HNS guidelines for diagnosis of Meniere’s disease on clinical presentation in patients presenting with episodic spinning vertigo spells lasting at least 30 minutes with asymmetric sensorineural hearing loss, not explained by other pathology, associated with either aural fullness or roaring tinnitus [2]. ENG and EVestG testing was performed after the clinical diagnosis was made.

Vestibular neuritis was diagnosed in patients presenting with a history predominantly of a prolonged spell of spinning vertigo, possibly with nausea and vomiting, but no hearing loss or other focal neurological signs or symptoms.

Benign positional vertigo was diagnosed in patients presenting with brief episodes of spinning vertigo precipitated by changes of the head with respect to gravity, lasting less than a minute and positive Dix-Hallpike test marked by symptomatic, paroxysmal, geotrophic, rotatory nystagmus, associated latency of onset after assuming the provocative position with no other pathology.

Non-specific dizziness was diagnosed in one patient who presented with episodic imbalance episodes lasting hours, occurring erratically, without hearing loss or evidence of other neuro-otological disorder on clinical exam.

### EVestG testing

The EVestG technique has been described previously [5]. Briefly, it consists of modified electocochleography (ECoG) [4] recording in which the multiple repetitions of acoustic stimuli of ECoG are replaced by a vestibular tilt stimulus, performed only once or twice instead of hundreds of times as for ECoG. Active electrode is placed inside ear canal and reference electrode on the ear lobe (as explained in the accompanying paper [5]). For this study, we considered the ipsilateral, contralateral (roll) tilt stimuli only; we will present the results of backward tilt stimulus in a future paper. For each tilt time, periods of interest were labeled at times of the positional changes in the tilt as illustrated in Figure two of the accompanying paper [5].

As explained in the accompanying paper [5], the algorithm was trained using a different dataset than the one in this study. Based on the outcome of that study, we calculated the same final characteristic features selected as those features in the training study; then, using linear discriminant analysis (LDA) [6] (trained by the previous dataset), we assigned a probability as the feature belonged to a person either with “Meniere’s” or “not Meniere’s”. In other words, each feature was used in a LDA classifier to vote for “Meniere’s” or “not Meniere’s”. Averaging the probabilities for each feature from the LDA classifiers, resulted in a final “vote”; if the average probability was equal or greater than 50% for the “Meniere’s” vote, the subject was then classified as Meniere’s disease.

### Statistical analysis

The sensitivity and specificity of EVestG testing as predictors of the clinical diagnosis of Meniere’s disease were determined by comparing the final vote of the classification results with those of the clinical diagnosis. Using IBM SPSS v22 (Chicago) Univariate ANOVA was carried out on the patients to determine whether other vestibular test results (unilateral weakness, sum of the four caloric tests) were predictive of the final EVestG-based classification; this was not run on control subjects as they had no ENG or audiometric data. Univariate ANOVA was also carried out on the combined patient and control subjects data to assess differences in the final EVestG-based classification among controls, Meniere’s patients and non-Meniere’s patients. We include information on the “effect size (η^2^), to estimate the amount of variability accounted for by the model, as well as statistical significance at the level of *p = 0.05*. Finally, linear regression analysis was carried out on the final EVestG-based classification and the mean SRT to assess the possibility whether the EvestG results were dependent on hearing.

## Results

The EVestG-based diagnostic analysis correctly identified Meniere’s disease in 3 of 4 Meniere’s patients (75% sensitivity) and correctly identified non-Meniere’s subjects (other dizziness diagnoses and controls) in 12 of 15 patients (80% specificity). The results are summarized in Table 1. If only patients are included, the sensitivity for detection of Meniere’s disease remains 75% but the specificity (3 out of 5 patients with non-Meniere’s diagnoses) would drop to 60%. The only Meniere’s patient identified incorrectly was the patient with the crisis of Tumarkin variant and had undergone intratympanic gentamicin ablation therapy suggesting that EVestG may only apply to classical Meniere’s disease.

**Table 1:** **Classification of subjects using EvestG probability >0.5 criterion**

	**Meniere’s**	**Non-Meniere’s**	**Totals**
**EvestG suggests Meniere’s**	3	3	6
**EvestG does not suggest Meniere’s**	1	12	13
**Totals**	4	15	19

Classification of subject based on EvestG testing final vote probability of Meniere’s disease >0.5 for Meniere’s, and Non-Meniere’s (other dizziness diagnosis plus Controls). In this sample the sensitivity of the test was 75% and the specificity was 80%. If controls are excluded the specificity remains 75% but the sensitivity drops to 60%.

Univariate analysis of variance (ANOVA) was conducted for vestibular tests. The final EVestG-based classification was the dependent variable and Meniere’s (yes or no), unilateral weakness score from ENG testing and sum of the four calorics were three independent variables. The skew test [7] indicated that the data did not depart significantly from a normal distribution justifying a parametric test. Levene’s test of equality of variances indicated that variances did not differ significantly so adjustment for unequal variances was not needed. There were no significant differences for EVestG-based classification for Meniere’s disease (yes or no) (*F* = 2.76, *p* = 0.16, η^2^ = 0.356), unilateral weakness score (*F* = 2.66, *p* = 0.164, η^2^ = 0.347) or sum of caloric tests *(F* = 0.153 *p* = 0.711, η^2^ = 0.030). Regression analysis was performed to assess the effect of hearing on final EVestG-based classification. The relationship between final EVestG-based classification e and mean SRT was not significant (*r*^2^ = 0.51, *p* = 0.055).

## Discussion

This paper illustrates the great difficulty in recruiting and testing volunteer patients, who may not be feeling well for clinical trials such as this. Although EVestG testing is safe, quick and free of adverse effects, subjects, particularly subjects who do not feel well, are reluctant to participate. The selection of subjects who are not acutely ill probably makes this test more applicable to the typical clinical situation for vestibular testing, usually performed when patients are asymptomatic. We started with 256 patients, which reduced to 9 in the final analysis. While we are encouraged by these preliminary results, we must point out that this is a small sample.

The roll stimulus was chosen to highlight unilateral pathologies. Meniere’s disease usually (and for all patients in this series) a unilateral disease so that it is not surprising that side tilts (roll) were diagnostic. With larger datasets we may find that the neural patterns that we identified herein are secondary to some other factors such as hearing loss, general vestibular hypofunction, or something else. The goal is to identify neural firing patterns that permit the objective, specific diagnosis of Meniere’s disease. We expect that greater understanding of the pathophysiology would follow, as would more reliable, measureable treatments. Nevertheless, we need larger numbers to validate the diagnostic ability of EVestG. We realize that many other factors and combinations of factors need to be assessed and controlled for but this requires larger numbers of patients to accomplish. Importantly, if the training of the classifier was to include other dizziness diagnosis improved separation of these pathologies from Meniere’s may be seen; this is a current investigation.

Diagnosis of Meniere’s disease is currently based on clinical criteria and the variability and subjectivity of clinical diagnosis is another source of error. In a larger dataset we anticipate that the disagreement between objective and subjective diagnoses will decrease as the standard deviations in each group decrease and the groups’ data form more defined clusters. Thus, with a larger dataset we hope to enhance the diagnostic classifier algorithm to become a useful tool to the level that clinicians will have enough confidence in EVestG to trust the objective diagnosis over clinical impressions. Efforts toward this goal are ongoing.

## Conclusion

EVestG testing is a sophisticated method of detecting signal features that may be useful in the objective diagnosis of Meniere’s disease.
